# LncRNA RBM5-AS1 Promotes Osteosarcoma Cell Proliferation, Migration, and Invasion

**DOI:** 10.1155/2021/5271291

**Published:** 2021-03-15

**Authors:** Biyong Deng, Runsang Pan, Xin Ou, Taizhe Wang, Weiguo Wang, Yingjie Nie, Houping Chen

**Affiliations:** ^1^The Orthopedic Surgery Department of Guizhou Province Orthopedic Hospital, China; ^2^The Orthopedic Surgery Department of Guiyang Maternal and Child Health-Care Hospital, Guiyang, Guizhou Province 550000, China; ^3^Department of Pathology of Guizhou Province Orthopedic Hospital, China; ^4^Science and Education Division of Guizhou Province People's Hospital, Guiyang, Guizhou Province 550000, China

## Abstract

**Purpose:**

Osteosarcoma (Os) is the most frequent malignant tumor of the bone in the pediatric age group, and accumulating evidences show that lncRNAs play a key role in the development of Os. Thus, we investigated the role of RBM5-AS1 and its molecular mechanism.

**Methods:**

The expression of RBM5-AS1 in Os tissues and cell lines was detected by real-time polymerase chain reaction (QPCR). The effect of RBM5-AS1 on the proliferation of Os cells was detected using CCK8 assays and flow cytometry. The effect of RBM5-AS1 on the migration and invasion of Os cells was detected by transwell assays. And we performed QPCR and western blotting assays to investigate the relationship between RBM5-AS1 and RBM5. Finally, western blotting assays were performed to explore the mechanism of RBM5.

**Results:**

LncRNA RBM5-AS1 was overexpressed in the Os tissues and cell lines. And lncRNA RBM5-AS1 promoted Os cell proliferation, migration, and invasion in vitro and tumor growth in vivo. LncRNA RBM5-AS1 targets RBM5 in Os cells.

**Conclusion:**

To sum up, the results showed that lncRNA RBM5-AS1 promotes cell proliferation, migration, and invasion in Os.

## 1. Introduction

Osteosarcoma (Os) is the most frequent malignant tumor of the bone in the pediatric age group [1], and the long-term survival rate for patients with localized Os is about 65%, whereas it is less than 20% for patients with metastatic Os [2]. The outcome in survival rates highlights the need for novel pathways and targets [3].

Long noncoding RNA (lncRNA) is a large class of RNA molecules with size larger than 200 nucleotides and without protein-coding capability [4]. The functions of lncRNAs range broadly from regulating chromatin structure and gene expression in the nucleus to control messenger RNA (mRNA) processing, mRNA posttranscriptional regulation, cellular signaling, and protein activity in the cytoplasm [5]. With growing numbers of lncRNAs being assigned to biological functions, the specificity of the lncRNA expression is recognized as biomarkers and the development of highly targeted therapeutics [6].

RBM5-AS1 is a nuclear-retained transcript that selectively interacted with *β*-catenin [7]. Mechanistic investigations showed that RBM5-AS1 activity is critical for the functional enablement of colon cancer stem-like cells [8], but its function in Os is still unclear. So, we investigated the RBM5-AS1 molecular mechanisms.

Then, we predicted that RBM5-AS1 regulates RBM5. RBM5 (previously referred to as g15, LUCA-15, and H37) is an RNA-binding protein that has the ability to modulate apoptosis [9]. RBM5 is a tumor suppressor gene, and it has been found that it is most frequently deleted at the earliest stage of lung cancer development [10, 11]. And in this article, we study the effect of RBM5-AS1 to RBM5.

## 2. Materials and Methods

### 2.1. Patients and Cell Lines

Os samples were acquired from the Department of Orthopedic Surgery, Guizhou Province People's Hospital (Guiyang, China). The study protocol was approved by the Animal Care Welfare Committee of the Guizhou Province Orthopedic Hospital. The human approval number is No. 20190706032. The human Os cell lines MG63, U2OS, SAOS2, HOS, 143B, and the normal osteoblast cell line hFOB1.19 were acquired from ATCC (American Type Culture Collection; Manassas, VA, USA). All cell lines were cultured at 37°C in an atmosphere containing 5% CO_2_ in DMEM or RPMI-1640 (Gibco, New York, USA) supplemented with 10% fetal bovine serum (Thermo Fisher Scientific, Massachusetts, USA).

### 2.2. Cell Transfection

Cells (2 × 10^5^) were seeded on the day before transfection with shRNAs (RiboBio, Guangzhou, China). Transfection was performed using lipofectamine 3000 (Life Technologies Co., Carlsbad, CA, USA) according to the manufacturer's instructions. We performed assays after 48 h. The sequence of RBM5-AS1 NC shRNA is 5′-GATTCTTCTTCTGTTCTGACATACT-3′, and the sequence of RBM5-AS1 shRNA is 5′-CCTTTCATTCTGAATTCATGTGCTT-3′. RBM5 upregulated and downregulated lentiviruses were purchased from GeneChem (Shanghai, China). All transfections were performed according to the manufacturer's instructions.

### 2.3. RNA Isolation, Reverse Transcription, and QPCR

Total RNA were extracted using TRIzol (Invitrogen, Carlsbad, CA, USA) reagent according to the manufacturer's instructions. Reverse transcription of lncRNAs was performed with PrimeScript RT Master Mix (Takara, Japan). QPCR was conducted using SYBR Green (Takara, Japan) according to the manufacturer's instructions. The results were normalized to the GADPH expression and calculated according to the 2^-*ΔΔ*Cq^ method. All experiments were in triplicate. Primer sequences were showed as follows: RBM5-AS1 forward sequence: 5′-TGGGAATGGGGAAGAGAAC-3, RBM5-AS1 reversed sequence: 5′-GGGAATAGTGTGTGGCAAAAG-3′; RBM5 forward sequence: 5′-GCACGACTATAGGCATGACAT-3′, RBM5 reversed sequence: 5′-AGTCAAACTTGTCTGCTCCA-3′.

### 2.4. Western Blotting

Total cell proteins were extracted using RIPA lysis buffer (Solabio, Beijing, China) containing protease and phosphatase inhibitors (Roche, Basel, Switzerland). Proteins (20 *μ*g) were resolved using 10% SDS polyacrylamide electrophoresis and electrotransferred to polyvinylidene difluoride (PVDF) membrane (Millipore, Bedford, MA). Western blots were probed with antibodies against RMB5 and GAPDH (1 : 1000, CST, USA) at 4°C overnight. After washing, the blots were then incubated with the secondary antibodies, goat anti-mouse (1 : 2000), and goat anti-rabbit (1 : 2000) (Boster Bio, USA) for 2 h at room temperature.

### 2.5. Colony Formation Assay

Transfected Os cells were planted into 6-well plates (1 × 10^3^ cells/plate) and incubated in DMEM or RPMI 1640 supplemented with 10% FBS at 37°C for 2 weeks. Then, cells were fixed in methanol for 30 minutes, stained with 1% crystal violet dye, and then counted. All experiments were performed with three independent trials.

### 2.6. Flow Cytometry

Transfected Os cells were planted into 6-well plates and cultured 2 days, and MG63 and 143B cells were fixed with 70% ethanol, respectively, and signed with propidium iodide (Sigma-Aldrich) in the dark (50 g/mL) for about 30 minutes in the presence of RNaseA (SigmaAldrich). And samples were detected by flow cytometry (Beckman, Cytomics FC 500, USA).

### 2.7. Xenograft Transplantation

Four weeks female nude mice (Beijing Huayi Kang Company, Beijing, China) were divided into two groups, three per group. MG63 cells were divided into NC and sh-RMB5-AS1 groups, 1 × 10^6^ of each was intraperitoneally injected, and the mice were observed each week and weighed. After 8 weeks, or when the nude mice died naturally, the surviving mice were killed using cervical dislocation, and tumors were removed and weighed. The animal ethical approval number is No. 20000584.

### 2.8. Transwell Migration and Invation Assays

Transwell invation assays were performed by transwell chambers (Corning- Costar; pore size 8 *μ*m) coated with Matrigel (Sigma); though, migration assays were without Matrigel. Transfected Os cells resuspended with FBS-free medium were added to the top chamber, and the medium with 10% FBS was filled in the bottom chamber as chemotaxin. After 48 h, we fixed and stained the cells. Cells from five random fields (×40 magnifications) were counted and photographed under the microscope.

### 2.9. Statistical Analysis

The Student *t-*test was performed to analyze the significance of differences between the mean values of three independent experiments. A significant difference was defined as *P* < 0.05. Results are displayed as mean ± SD.

## 3. Results

### 3.1. LncRNA RBM5-AS1 Was Overexpressed in the Os Tissues and Cell Lines

**Figure 1:**
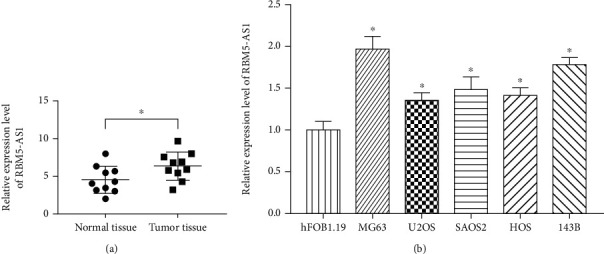
LncRNA RBM5-AS1 was overexpressed in the Os tissues and cell lines. (a) lncRNA RBM5-AS1 was significantly upregulated in Os tissues compared with normal tissues. (b) lncRNA RBM5-AS1 was significantly upregulated in Os cell lines compared with hFOB1.19 cells by RT-PCR. ^∗^*P* < 0.05.

To investigate the role of LncRNA RBM5-AS1 in the Os, the expression of RBM5-AS1 between normal tissues and Os tissues was detected. The results showed that the expression of LncRNA RBM5-AS1 was significantly upregulated in the Os tissues compared with normal tissues (Figure 1(a), *P* < 0.05). And the expression among different Os cell lines and normal cells was also detected through QPCR. As shown in Figure 1(b), the results made significantly differences, and the expression of RBM5-AS1 was overexpressed in the Os cells.

### 3.2. Downregulated lncRNA RBM5-AS1 Inhibited Os Cells Proliferation In Vitro

**Figure 2:**
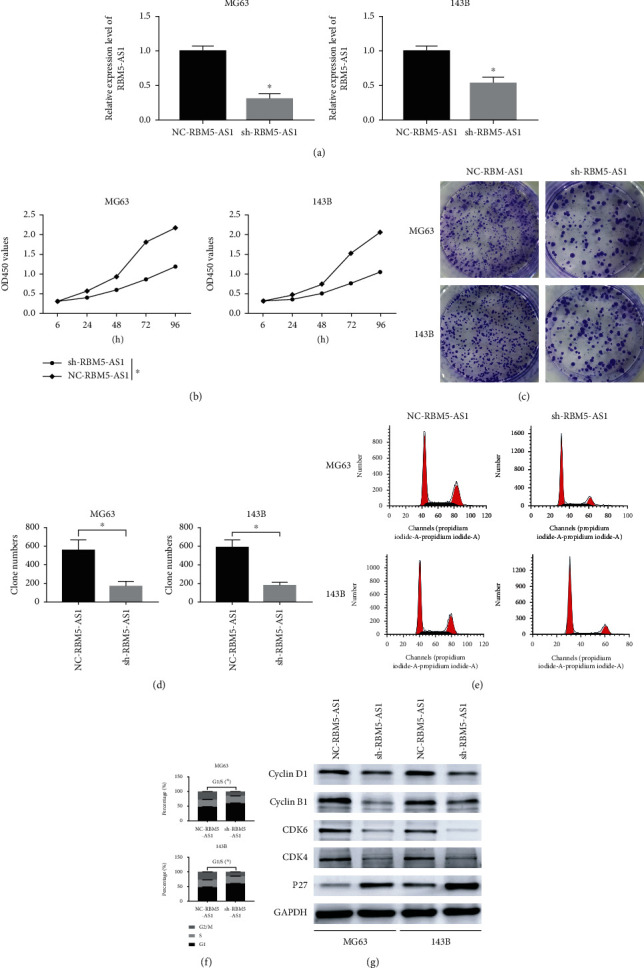
LncRNA RBM5-AS1 promoted Os cells proliferation in vitro. (a) Stable lncRNA RBM5-AS1 knockdown in MG63 and 143B cells was confirmed by RT-PCR. (b) CCK8 assays showed the effect of RBM5-AS1 on the proliferation of MG63 and 143B cells. (c, d) Colony formation assays showed a significantly lower proliferative rate in both cell lines following RBM5-AS1 knockdown. (e, f) Flow cytometry was used to explore whether RBM5-AS1 promoted Os cell proliferation. (g) Western blotting was used to detect the protein level of cyclin B1, cyclin D1, CDK4, and CDK6 after RBM5-AS1 inhibition.

To investigate the function of lncRNA RBM5-AS1 in Os cells, the RBM5-AS1 was downregulated by transfecting lentivirus. And the cells were divided into the negative control (NC-RBM5-AS1) group and sh-RBM5-AS1 group. As shown in Figure 2(a), the expression of RBM5-AS1 in the sh-RBM5-AS1 group was downregulated. And CCK8 results showed that downregulated RBM5-AS1 inhibited the Os cell proliferation (Figure 2(b)). And the colony formation assays displayed sh-RBM5-AS1 groups reduced the capability of colony formation (Figures 2(c) and 2(d)). Then, we explored how lncRNA RBM5-AS1 effect the cell cycle by flow cytometry, and the results indicated that the fraction of cells in G1 phase increased, while the fraction of cells in the S phase decreased compared with the NC-RBM5-AS1 group (Figures 2(e) and 2(f)). Western blot analysis showed that sh-RBM5-AS1 groups reduced cyclin B1, cyclin D1, CDK4, and CDK6 which indicated that downregulated RBM5-AS1 promoted cell cycle progression (Figure 2(g)).

### 3.3. Downregulated lncRNA RBM5-AS1 Inhibited Os Cells Migration and Invasion In Vitro

**Figure 3:**
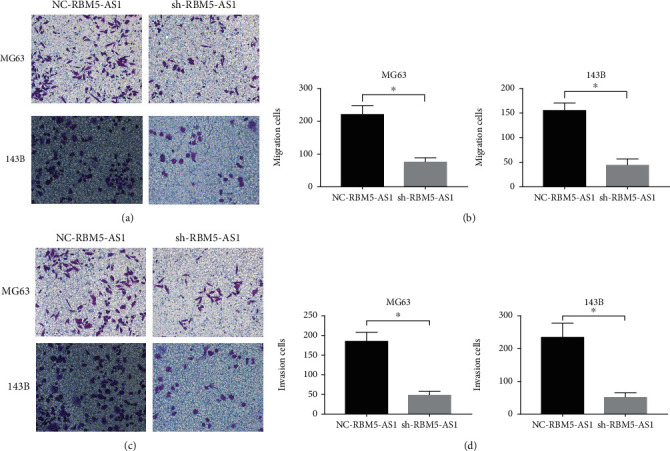
LncRNA RBM5-AS1 promoted Os cell migration and invasion in vitro. (a, b) Transwell assays showed significantly decreased migration abilities in MG63 and 143B cells while RBM5-AS1 knockdown. (c, d) Transwell assays showed significantly decreased invasion abilities in both cell lines while RBM5-AS1 knockdown. ^∗^*P* < 0.05.

We also performed the transwell assays to detect the ability of migration and invasion. The results showed that the migration and invasion cells in the NC-RBM5-AS1 groups were larger compared to the sh-RBM5-AS1 groups (Figures 3(a)–3(d)).

### 3.4. Downregulated lncRNA RBM5-AS1 Inhibited Os Tumor Growth In Vivo

**Figure 4:**
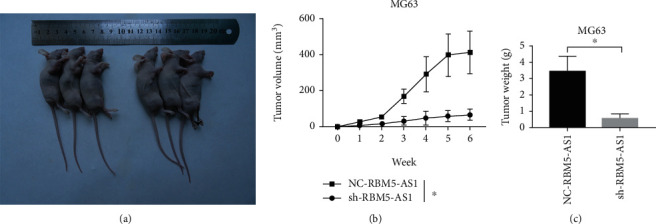
lncRNA RBM5-AS1 promoted Os tumor growth in vivo. (a) The results showed that lncRNA RBM5-AS1 promoted Os cell tumor growth in vivo. (b, c) The RBM5-AS1 knockdown group showed reduced tumor volumes and weight compared with controls. ^∗^*P* < 0.05.

We established subcutaneous xenograft models to explore the effect of lncRNA RBM5-AS1. And the tumors in NC- RBM5-AS1 groups were significantly larger than that in the sh-RBM5-AS1 group (Figure 4(a)). In addition, the tumor volume and weight decreased significantly in the sh-RBM5-AS1 group (Figures 4(b) and 4(c)).

### 3.5. lncRNA RBM5-AS1 Targets RBM5 in Os Cells

**Figure 5:**
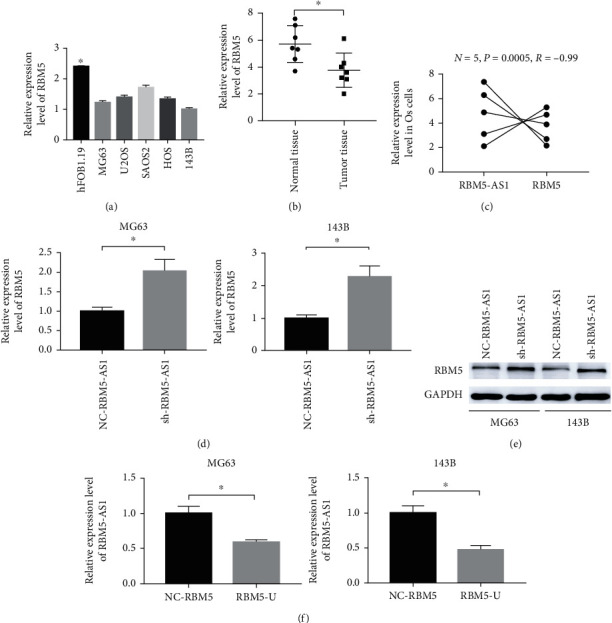
lncRNA RBM5-AS1 targets RBM5 in Os. (a, b) RT-PCR showed that the RBM5 expression was markedly reduced in Os cell lines and tissues. (c) The correlation between the RBM5-AS1 and RBM5 expression in Os cells. (d, e) The results showed that the RBM5 expression was markedly reduced in NC- RBM5-AS1 cells by RT-PCR and western blot. (f) The results showed that the RBM5-AS1 expression was markedly reduced in RBM5-U cells by RT-PCR.

Since lncRNA RBM5-AS1 is an antisense lncRNA of RBM5, we further investigated if RBM5 is a functional target of lncRNA RBM5-AS1. The relative expression of RBM5 in Os cell lines and tissues was detected by Qpcr, and the results showed that RBM5 was downregulated (Figures 5(a) and 5(b)). And Figure 5(c) shows an opposite relationship between lncRNA RBM5-AS1 and RBM5 expression in Os cell lines. To further explore the interaction between lncRNA RBM5-AS1 and RBM5, we inhibited the expression of lncRNA RBM5-AS1, and the results showed that the expression of RBM5 was increased (Figures 5(d) and 5(e)). Next, we upregulated the expression of RBM5, and the expression of lncRNA RBM5-AS1 was decreased (Figure 5(f)).

### 3.6. The Downregulated RBM5 Expression Could Recover the Inhibition of RBM5-AS1 Knockdown in Os Cell Lines

**Figure 6:**
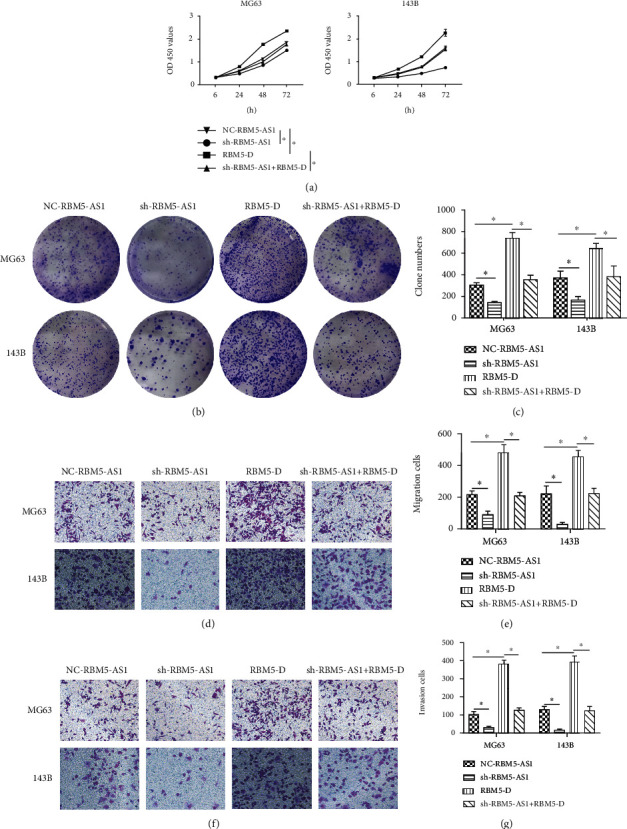
The downregulated RBM5 expression could recover the inhibition of RBM5-AS1 knockdown in Os cell lines. (a) The CCK8 assay was used to detect the proliferation of the NC-RBM5-AS1 group, sh-RBM5-AS1 group, RBM5-D group, and sh-RBM5-AS1+RBM5-D group, and the results showed that cells in the sh-RBM5-AS1+RBM5-D group rescued the inhibitory effect of sh-RBM5-AS1. (b, c) Cells in the sh-RBM5-AS1+RBM5-D group rescued the inhibitory effect of sh-RBM5-AS1 on colony formation. (d)–(g) Cells in the sh-RBM5-AS1+RBM5-D group rescued the inhibitory effect of sh-RBM5-AS1 on Os cell migration and invasion.

RBM5-AS1 was overexpressed in Os tissues and cells, but RBM5 was downregulated. To verify the function of RBM5 in Os progression, the Os cells were divided into the NC-RBM5-AS1 group, sh-RBM5-AS1 group, RBM5 downregulated group (RBM5-D group), and sh-RBM5-AS1+RBM5-D group. As the results showed, the sh-RBM5-AS1+ RBM5-D group rescued the inhibition of sh-RBM5-AS1 on cell proliferation (Figure 6(a)) and colony formation (Figures 6(b) and 6(c)). These results indicated that the downregulated RBM5 expression could recover the inhibition of RBM5-AS1 in Os cell lines. Furthermore, transwell assays indicated that the sh-RBM5-AS1+ RBM5-D group rescued the inhibition of sh-RBM5-AS1 on cell migration and invasion (Figures 6(d)–6(g)).

## 4. Discussion

Os is the most frequent malignant tumor of the bone in the pediatric age group, and the long-term survival rate for patients with localized Os is about 65%, whereas it is less than 20% for patients with metastatic Os.

Accumulating evidence suggests that long noncoding RNA plays crucial roles in the progression of various cancers including regulation of gene expression, imprinting, chromatin modification, transcription, and posttranslational processing [8, 12]. Li et al. generalized that many lncRNAs were important regulators for malignancies [13]. Zhou et al. investigated that lncRNA SNHG12 promoted tumorigenesis and metastasis in Os [14]; He et al. found that the lncRNA LINC00628 overexpression inhibited the growth and invasion through regulating the PI3K/Akt signaling pathway in Os [15]. In this article, we explored the effect of RBM5-AS1 in Os. Until now, there were few researches of RBM5-AS1 [8]. There has been reported that RBM5-AS1 participated in fracture healing and inhibited apoptosis of bone cells through the upregulation of *β*-catenin [7]. Li et al. studied that long noncoding RNA RBM5-AS1 promoted the aggressive behaviors of oral squamous cell carcinoma by regulating the miR-1285-3p/YAP1 axis [16]. In the present study, we demonstrated that RBM5-AS1 was significantly increased in Os tissues and cell lines. In addition, knockdown of RBM5-AS1 significantly inhibited proliferation, migration, and invasion of Os cells in vitro as well as tumor growth in vivo.

Therefore, these results indicated that RBM5-AS1 promoted the progression of Os. And we predicted that RBM5-AS1 acted on RBM5 because RBM5-AS1 was the antisense lncrna of RBM5. RBM5, an RNA-binding protein, was reported as a tumor suppressor gene. Jiang et al. investigated that RBM5 inhibited tumorigenesis of gliomas through inhibition of Wnt/*β*-catenin signaling and induction of apoptosis [17]. Shao et al. reported that the tumor suppressor gene RBM5 inhibited lung adenocarcinoma cell growth and induced apoptosis [18]. And we found that RBM5 can rescue the inhibitory effect of RBM5-AS1 on Os cells. These results showed that RBM5-AS1 targeted RBM5, but the underlying mechanism is still unclear, and we can further research it.

## Data Availability

The data used to support the findings of this study are available within the article.
